# Retraction Note: A switch in the poly(dC)/RmlB complex regulates bacterial persister formation

**DOI:** 10.1038/s41467-019-11189-7

**Published:** 2019-07-11

**Authors:** Xu Chen, Gen Li, Xuewei Liao, Jie Fang, Bo Li, Shanshan Yu, Mingming Sun, Jun Wu, Lihao Zhang, Yi Hu, Jiaguo Jiao, Ting Liu, Li Xu, Xiaoyun Chen, Manqiang Liu, Huixin Li, Feng Hu, Kouhong Sun

**Affiliations:** 1 Soil Ecology Lab, College of Resources and Environmental Sciences, Nanjing Agricultural University, Nanjing, China; 2 Center for Analysis and Testing, Nanjing Normal University, Nanjing, China; 3 Jiangsu Collaborative Innovation Center for Solid Organic Waste Resource Utilization, Nanjing, China; 4 Jiangsu Key Laboratory for Solid Organic Waste Utilization, Nanjing, China; 5 Zoonbio Biotechnology Co., Ltd, Nanjing, China

**Keywords:** Bacteria, Microbial genetics, Policy and public health in microbiology

Retraction Note: *Nature Communications* 10.1038/s41467-018-07861-z, published online 3 January 2019.

The authors are retracting this Article as we are unable to reproduce all of the original findings in the paper except those relating to an increase in persister levels in wild-type *Pseudomonas aeruginosa*. At this time we are unable to elucidate the specific explanation for this, however, this finding does now undermine our full confidence in the conclusions presented in this study and we feel it is best to retract the Article at this stage. We apologise for any inconvenience this may have caused to the readers.

All the authors agree with the retraction.

